# *Bacillus* Cyclic Lipopeptides Iturin and Fengycin Control Rice Blast Caused by *Pyricularia oryzae* in Potting and Acid Sulfate Soils by Direct Antagonism and Induced Systemic Resistance

**DOI:** 10.3390/microorganisms9071441

**Published:** 2021-07-03

**Authors:** Van Bach Lam, Thibault Meyer, Anthony Arguelles Arias, Marc Ongena, Feyisara Eyiwumi Oni, Monica Höfte

**Affiliations:** 1 Laboratory of Phytopathology, Department of Plants and Crops, Faculty of Bioscience Engineering, Ghent University, Coupure Links 653, B-9000 Ghent, Belgium; van.lambach@ugent.be (V.B.L.); feyisara.oni@univ-reims.fr (F.E.O.); 2 Microbial Processes and Interactions Unit, Faculty of Gembloux Agro-Bio Tech, University of Liège, B-5030 Gembloux, Belgium; thibault.meyer@univ-lyon1.fr (T.M.); aarguelles@uliege.be (A.A.A.); marc.ongena@uliege.be (M.O.)

**Keywords:** *Bacillus altitudinis*, *Bacillus velezensis*, fengycin, iturin, surfactin, rice blast, *Pyricularia oryzae*, acid sulfate soil

## Abstract

Rice monoculture in acid sulfate soils (ASSs) is affected by a wide range of abiotic and biotic constraints, including rice blast caused by *Pyricularia oryzae.* To progress towards a more sustainable agriculture, our research aimed to screen the biocontrol potential of indigenous *Bacillus* spp. against blast disease by triggering induced systemic resistance (ISR) via root application and direct antagonism. Strains belonging to the *B. altitudinis* and *B. velezensis* group could protect rice against blast disease by ISR. UPLC–MS and marker gene replacement methods were used to detect cyclic lipopeptide (CLiP) production and construct CLiPs deficient mutants of *B. velezensis*, respectively. Here we show that the CLiPs fengycin and iturin are both needed to elicit ISR against rice blast in potting soil and ASS conditions. The CLiPs surfactin, iturin and fengycin completely suppressed *P. oryzae* spore germination resulting in disease severity reduction when co-applied on rice leaves. In vitro microscopic assays revealed that iturin and fengycin inhibited the mycelial growth of the fungus *P. oryzae*, while surfactin had no effect. The capacity of indigenous *Bacillus* spp. to reduce rice blast by direct and indirect antagonism in ASS conditions provides an opportunity to explore their usage for rice blast control in the field.

## 1. Introduction

Acid sulfate soils (ASSs) account for more than 1.7% of the global cultivated land worldwide and are distributed over as much as 24 million hectares [1,2]. Most of these soils are cultivated with rice because of its tolerance to acidic conditions [3]. In Vietnam, more than 1.6 million hectares of ASSs in the Mekong Delta have been ameliorated for rice cultivation by managing submergence or drainage, applying phosphate fertilizer and lime, and using resistant cultivars [4,5]. Rice monoculture in ASSs has recently been intensified with three cropping seasons per year, instead of the single or double cropping seasons of the past decades. This leads to a higher disease pressure, resulting in a drastic reduction in rice yield [6,7,8]. A prevailing disease which ravages rice fields and reduces annual rice production, especially in Vietnam, is the foliar rice blast disease caused by *Pyricularia oryzae* (syn: *Magnaporthe oryzae*). *P. oryzae* is a hemibiotrophic pathogen with biotrophic and necrotrophic growth stages [9,10]. The penetration of this pathogen is initiated by asexual spores which attach to the leaf surface and germinate in high humidity within four to six hours [11]. Appressoria, melanin pigmented unicellular structures [9], are formed after germination and the extension of a short germ tube (15–30 µm), and penetrate the leaf surface by mechanical force [11]. Blast disease spreads and becomes more serious in favorable environmental conditions, such as a high relative humidity, 26–28 °C air temperature, and susceptible rice cultivars. Therefore, rice blast is especially severe in Vietnam because of its humid and tropical climate conditions [12]. In order to curtail this disease, farmers have resorted to the excessive use of fungicides, leading to pathogen resistance. As a result, chemical fungicides are gradually becoming inoperative. Therefore, the use of bacterial biocontrol agents to control rice blast is gaining attention [13,14,15,16,17,18,19,20,21,22,23].

Among plant growth promoting rhizobacteria (PGPR) strains, *Pseudomonas* and *Bacillus* spp. have been explored for induced systemic resistance (ISR) to rice pathogens [24,25]. *Bacillus* isolates are good candidates for biological control in view of their unique characteristics, including metabolite production with antagonistic activity against diverse phytopathogens, ease of formulation, and a high viability compared with vegetative cells [26]. They are capable of forming endospores that are highly resistant to adverse environment conditions and can easily be applied in farming systems [27]. *Bacillus* spp. are known to produce five different cyclic lipopeptide families, namely, fengycins/plipastatins, surfactins, iturins, kurstakins [28] and locillomycins [29]. Especially, *Bacillus* isolates belonging to *B. velezensis, B. thuringiensis* and *B. pumilus* have been reported as valuable biocontrol agents against diverse phytopathogens through the production of cyclic lipopeptides (CLiPs) [15,30,31,32]. CLiPs, which are amphiphilic molecules, are bioactive compounds that can control plant pathogens by both direct antagonism or via ISR [25,33].

In previous work, *B. subtilis* BBG111, which produces both surfactin and fengycin, and *B. subtilis* RFB104, which synthesizes surfactin and mycosubtilin, could elicit ISR against rice sheath blight caused by *Rhizoctonia solani*, but not against rice blast caused by *P. oryzae* [31]. He et al. [15] showed that cell-free supernatants of *B. subtilis* BJ-1 could suppress *P. oryzae* via both direct and indirect mechanisms, but they did not demonstrate the effects of individual metabolites/compounds (fengycin, surfactin, subtilin and bacilysin) in disease control. The genome of *B. velezensis* GA1 (previously called *B. subtilis* GA1) has been studied, demonstrating the antimicrobial potential of the strain due to its secondary metabolite diversity comprising surfactin, iturin A, fengycin and the siderophore bacillibactin [34]. To the best of our knowledge, no study has been done to investigate the capacity of *Bacillus* strains to induce resistance against rice blast under adverse soil conditions such as acid sulfate soils or in direct antagonism against *P. oryzae* spores using mutants impaired in CLiP production. 

Thus, the goal of our study was: (i) to test the ISR capacity of indigenous *Bacillus* spp. isolated from the rice rhizosphere in ASSs in Vietnam, hypothesizing that these strains might be best adapted to the harsh conditions encountered in these soils; (ii) to compare the ISR capacity of *B. velezensis* strains GA1 and RHF4.1–25, a rice rhizospheric isolate from acid sulfate soils in Vietnam; (iii) to investigate the role of surfactin, fengycin and iturin in ISR and direct antagonism against rice blast using single and double CLiP mutants of *B. velezensis* GA1. The novelty of this work is that we demonstrated the capacity of indigenous *Bacillus* spp., found in the rhizosphere of rice grown in ASSs, to control rice blast disease. Moreover, we could show the power of using mutants impaired in CLiP production to determine the role of these metabolites in biocontrol. 

## 2. Materials and Methods

### 2.1. Plant Material, Strains, Media and Growth Conditions

Rice (*Oryza sativa* L.) *indica* cv. CO39 was used as the plant material for bioassays in this study. For experiments in ASSs, the rice cultivar Jasmine 85 (a local rice variety in Vietnam) was used. Rice seeds were prepared as described previously [24,25]. Rice seeds were dehusked and dipped for 20 min in 2% (w/v) sodium hypochlorite solution at room temperature. Seeds were washed several times with sterile water, air dried and pregerminated for 2 days in Petri dishes containing sterile moistened filter paper at 28 °C in dark conditions. Subsequently, two day old rice seedlings were placed under greenhouse conditions with a 12 h light photoperiod at 30 ± 4 °C for 2 days to use for the experiment. Rice plants were maintained in a rice room (photoperiod with 12 h light; 30 ± 4 °C) afterwards. *Bacillus* isolates used in this study are listed in Table 1. The *Bacillus* isolates RHF4.1–25, RHF4.1–26, RHF3.1–20 and RHF2.1–7 originate from the rhizosphere of rice plants grown in ASSs in Vietnam. *B. velezensis* GA1 (formerly *B. subtilis* GA1) is a well-studied biocontrol agent that was originally isolated from strawberry fruit [35]. All *Bacillus* isolates were cultured on Luria–Bertani (LB) agar at 28 °C for 24 h. Broth cultures of *Bacillus* strains were cultured in LB broth at 150 revolutions per minute (rpm) and 28 °C for 24 h. *P. oryzae* VT5M1 [12] was maintained on complete medium (CM) plates [36] at 28 °C for 5–8 days.

### 2.2. Construction of Bacillus velezensis GA1 Mutants

*Bacillus velezensis* GA1 mutants deficient in the production of a single CLiP were constructed by marker gene replacement, as previously described [37]. To construct CLiP double mutants, a recombinant fragment containing a phleomycin cassette flanked by 1000 bp of the upstream region and 1000 bp of the downstream region of the targeted gene was generated by overlap PCR with specific primers (Table A1) (see Appendix A) [37]. The recombinant fragment was introduced into *B. velezensis* GA1 derivative mutants (GA1∆*srfaA*, GA1∆*ituA*, GA1∆*fenA*) by inducing natural competence, as previously described [37]. Gene replacement was selected by phleomycin resistance on LB medium. Gene deletions were confirmed by PCR analysis realized with the corresponding UpF and DwR specific primers (Table A1).

### 2.3. Preparation of Cell-Free Supernatants and UPLC–MS for Detection of CLiPs

To obtain cell-free culture filtrates of each isolate, a colony was transferred into 5 mL LB broth and incubated at 28 °C, 150 rpm for 24 h, then cultures were centrifuged at 13,000× *g* for 10 min. Subsequently, supernatants were filter sterilized through a Millipore filter (Millex-GV, 0.22 µm) to exclude bacterial cells. Cell free supernatants were collected for UPLC–MS analysis to detect and quantify CLiPs, as previous described [37], and utilized for direct antagonism assays in this study.

### 2.4. Potential of Indigenous Bacillus Strains to Trigger ISR against Rice Blast Caused by P. oryzae in Potting and Acid Sulfate Soil

Experiments performed to screen the ISR capacity of indigenous *Bacillus* spp. against rice blast caused by *P. oryzae* VT5M1, in both potting and ASS conditions, were conducted as described previously [24,25]. Briefly, for the preparation of bacterial inoculum, 15 mL of *Bacillus* seed culture was grown in 50 mL LB broth for 24 h at 28 °C. The amount of bacterial cell suspension was applied based on 600 g of sterile potting soil (Structural; Snebbout, Kaprijke, Belgium) or 800 g of sterile ASSs (a natural soil taken from representative ASSs in Kien Giang province, Vietnam), and adjusted to a final density of 1 × 10^7^ CFU g^−1^. S-methyl 1,2,3-benzothiadiazole-7-carbothioate (BTH) (Syngenta Crop Protection, Brussels, Belgium), a salicylic acid analogue that is known to induce resistance against rice blast [25], was included as a positive control at a concentration of 25 µM. BTH (stock solution of 50 mM) and cell suspensions were diluted in 100 mL of water and mixed with 600 g of potting soils or 800 g of ASS for 4 min. Healthy and diseased control treatments received the same amount of tap water. Roots of rice seedlings were inoculated with the standardized bacterial inoculum by soaking for 10 min before planting them in plastic trays (23 cm × 16 cm × 6 cm) containing inoculated potting soil or acid sulfate soil. Then, bacteria were applied as soil drench 3 days before inoculation with *P. oryzae* VT5M1. Plants were watered every two days and trays were weekly supplied with 200 mL of nutrient solution [FeSO_4_; 2 g/L and (NH_4_)_2_SO_4_; 1 g/L]. Pathogen inoculation was performed as described previously [25]. Spores of 8 day old *P. oryzae* VT5M1 [12] were dispersed in 0.5% (*w*/*v*) gelatin to obtain a final concentration of 5 × 10^4^ spores mL^−1^. One mL of spore solution was uniformly sprayed on each plant by using an airbrush compressor (Badger Airbrush model 150TM). Inoculated plants were placed for 22–24 h in a growth chamber in the dark (relative humidity ≥ 90%; 27 ± 5 °C), and further incubated in a greenhouse for disease development. Disease rating was performed 6 days after inoculation by counting the number of sporulating lesions on the youngest unfolded leaves. Photos of representative disease symptoms were taken after disease evaluation. For each treatment, three replicates of 7 plants (potting soils) or 5 plants (ASSs) each were used.

### 2.5. Role of Cyclic Lipopeptides in ISR Triggered by B. velezensis against P. oryzae in Potting and Acid Sulfate Soil Conditions

Based on the results of previous experiments, the most effective *Bacillus* isolate, namely, *B. velezensis* RHF4.1–25, was selected for further use and its efficacy was compared with *B. velezensis* GA1. In these assays cyclic lipopeptide mutants of *B. velezensis* GA1 were also included. Single mutants (∆*srfaA*, ∆*ituA* and ∆*fenA*) were tested in both potting and ASSs. Complementation of ∆*ituA* and ∆*fenA* isolates was carried out in potting soil by combining each isolate at half dose. Based on consistent results obtained in both acid sulfate soils and potting soils, and because of the limited availability of the acid sulfate soil, assays with double mutants (∆*srfaA-ituA*, ∆*srfaA-fenA* and ∆*fenA-ituA*) were only performed in potting soils. Bacterial inoculum preparation, experimental set up, disease infection, and evaluation were carried out in a similar manner as were performed in the assay above. For all experiments, treatments were performed in three replications, each comprising of 7 plants (potting soil) or 5 plants (ASSs).

### 2.6. Root Colonization Assay

Root colonization was evaluated as described previously [25,38]. At the time of disease rating, five rice roots per treatment were randomly chosen and gently washed in water to remove soil. The roots were weighed after air-drying for 2 min. Subsequently, roots were ground in 10 mL sterile saline (0.85% sodium chloride, *w*/*v*) and sterile sand by using a mortar and pestle. Following this, 100 µL of serially diluted suspensions were plated on LB agar and incubated for 24 h at 28 °C before counting *Bacillus* colonies based on their morphological characteristics. The data were log10 transformed prior to statistical analysis.

### 2.7. In Vitro Antagonism against P. oryzae Using Cell-Free Supernatants of B. velezensis Strains and GA1 CLiP Mutants

To investigate the effect of cell-free supernatants on the growth of *P. oryzae* mycelium, in vitro tests were carried out using plastic slides with five replications of each treatment. Sterile microscopic plastic slides covered with a thin, flat layer of water agar (Difco Bacto agar; BD Diagnostics, Le Pont-deClaix, France) were placed in a plastic Petri dish containing sterile, moist absorbent paper [39]. Subsequently, an agar plug (diameter = 8 mm) taken from an actively growing Complete Media (CM) plate of *P. oryzae* VT5M1 was inoculated at the center of each plastic slide. Two droplets (15 µL each) of a cell-free supernatant of the isolates were placed on two sides of the plastic slide (about 2 cm from the *P. oryzae* plug) while for the control treatment, two droplets of LB broth were used on both sides of the plug. All plates were incubated at 28 °C for five days. Microscopic slides were assessed by using an Olympus BX51 Microscope. Additionally, the diameter of mycelial growth was determined, and converted to the percentage growth inhibition based on the following formula:

$$\frac{(\mathrm{Growth}\;\mathrm{diameter}\;\mathrm{of}\;\mathrm{untreated}\;\mathrm{control}\;-\;\mathrm{Growth}\;\mathrm{diameter}\;\mathrm{of}\;\mathrm{treated}\;\mathrm{control})\times 100}{\mathrm{Growth}\;\mathrm{diameter}\;\mathrm{of}\;\mathrm{untreated}\;\mathrm{control}}$$

### 2.8. Influence of Cell-Free Culture Filtrates on P. oryzae Spore Germination and Appressoria Formation

For this experiment, filter sterilized supernatants were derived from strains RHF4.1–25, GA1 and its CLiP mutants (Table 1). Spore suspensions were diluted to obtain a final concentration of 5 × 10^4^ spores mL^−1^ and mixed with 100 µL of *Bacillus* cell-free supernatants to get a final concentration of 50% (*v*/*v*). The same amount of LB broth was used for the control treatment. Subsequently, a plastic slide (Fisher Scientific, Merelbeke, Belgium) containing fifty µL of the mixture was incubated in the dark at 28 °C. After a 4 h incubation period, spore germination was recorded by counting the number of germination tubes. Eight hours post incubation (hpi), fifty randomly selected spores were evaluated for appressoria formation. An Olympus BX51 Microscope was used to observe spore germination and appressoria formation. The assay was conducted twice.

### 2.9. Direct Effect of Cell-Free Supernatants to Reduce Rice Blast Symptoms Caused by P. oryzae Spores

To obtain 4 week old rice plants for disease infection, rice seedlings were planted in sterile potting soils (600 g per tray) and maintained under controlled conditions. Spore suspensions of *P. oryzae* VT5M1 were prepared as previously described [12]. Spores obtained from 8-day old *P. oryzae* VT5M1 cultures were added into 0.5% (*w*/*v*) gelatin to obtain a concentration of 5 × 10^4^ spores mL^−1^. One mL of cell-free supernatants was mixed with 1 mL of spore suspension to secure a final concentration of 50% (*v*/*v*). A compressor-powered air brush gun was used to spray the mixture onto rice plants (1 mL of the mixture per plant) and the trays were frequently rotated during this spraying process. The healthy treatment was sprayed with a mixture of 0.5% gelatin suspension and the same amount of sterile water, whereas the diseased treatment received a mixture of 0.5% gelatin and 5 × 10^4^ spores mL^−1^. Pathogen inoculation and disease assessment were performed as depicted above. Treatments were performed in three replications, each comprising of 7 plants. 

### 2.10. Statistical Data Analysis

The data of all experiments were statistically analyzed using the software package SPSS 25.0. To compare mean values among treatments, univariate ANOVA followed by Duncan’s post hoc tests were used and results had statistically significant differences when *p* < 0.05.

## 3. Results

### 3.1. Potential of Indigenous Bacillus Strains to Control Rice Blast by ISR in Potting Soil and ASS Conditions

**Figure 1:**
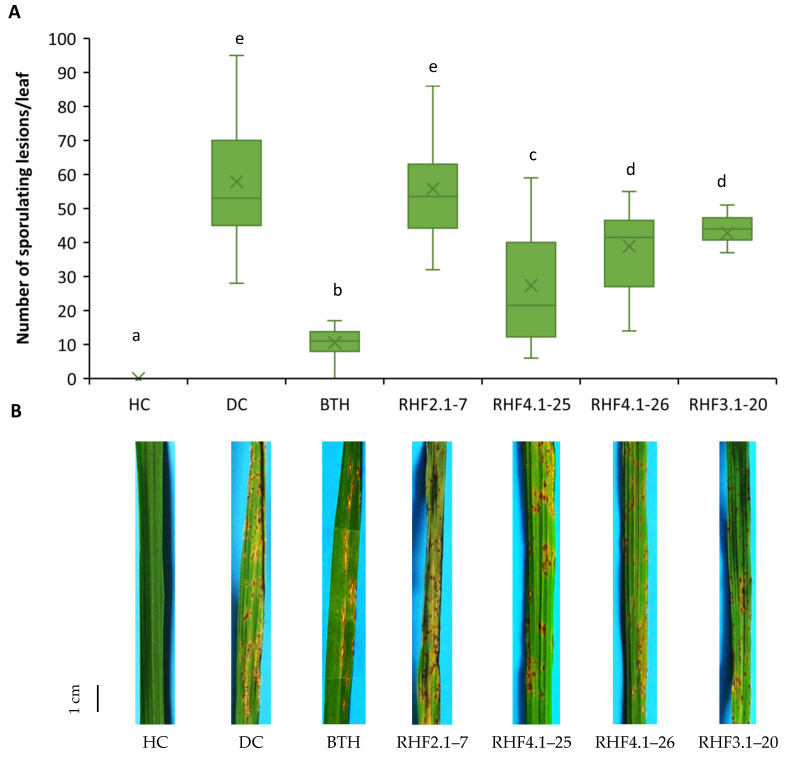
(**A**) Potential of *Bacillus* spp. isolated from the rice rhizosphere in acid sulfate soils to induce systemic resistance against *P. oryzae* in rice (variety CO39) grown in potting soil. Bacteria were applied by inoculating the roots of rice seedlings before transplanting and by a soil drench three days before pathogen inoculation. Four-week old rice plants were inoculated with 5 × 10^4^ spores mL^−1^ of *P. oryzae* VT5M1. Disease was evaluated six days post pathogen inoculation by counting the number of susceptible lesions. The experiment was carried out once in three repetitions, with seven plants each. Data are presented as boxplots (*n* = 21). One way ANOVA followed by Duncan’s post hoc tests were used and different letters among these treatments indicate statistically significant differences (*p* < 0.05). HC: healthy control; DC: diseased control; RHF2.1–7: *B. marisflavi*; RHF4.1–25: *B. velezensis*; RHF4.1–26 and RHF3.1–20: *B. altitudinis*; BTH: S-methyl 1,2,3-benzothiadiazole-7-carbothioate. (**B**) Representative pictures of disease symptoms taken at the time of disease evaluation. The scale bar represents 1 cm.

The four indigenous *Bacillus* strains used in this study were obtained from ASSs in Vietnam. Full details about their isolation, taxonomy and metabolic profile will be published elsewhere. Initially, they were tested for their ability to trigger ISR against rice blast disease in potting soil conditions. Three representative isolates belonging to *B. velezensis* (RHF4.1–25) and *B. altitudinis* (RHF4.1–26 and RHF3.1–20) could trigger ISR against rice blast caused by *P. oryzae* VT5M1, while *B. marisflavi* RHF2.1–7 was not effective (Figure 1). The most effective strain in triggering ISR was *B. velezensis* RHF4.1–25. The two isolates belonging to *B. altitudinis* were significantly less effective than *B. velezensis* RHF4.1–25. All tested *Bacillus* isolates effectively colonized the rice roots with densities ranging from 7.70 to 8.41 log CFU g^−1^ of fresh root (Table 2).

**Figure 2:**
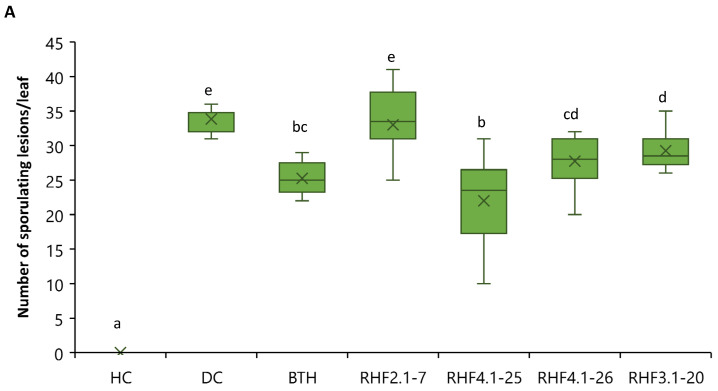

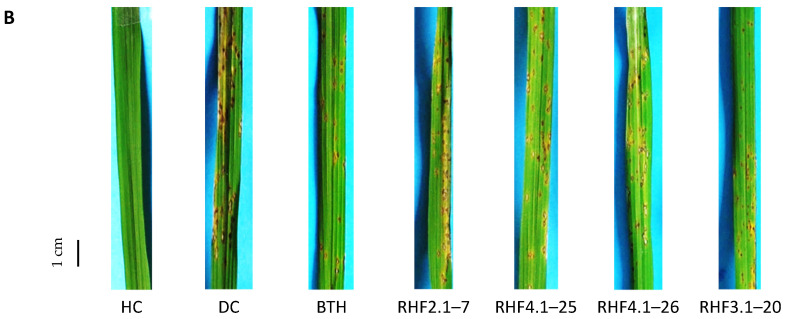
(**A**) Potential of *Bacillus* spp. isolated from the rice rhizosphere in acid sulfate soils to induce systemic resistance against *P. oryzae* in rice (variety Jasmine 85) grown in **acid sulfate soil**. Bacteria were applied by inoculating the roots of rice seedlings before transplanting and by a soil drench three days before pathogen inoculation. Four-week old rice plants were inoculated with 5 × 10^4^ spores mL^−1^ of *P. oryzae* VT5M1. Disease was evaluated six days post pathogen inoculation by counting the number of susceptible lesions. The experiment was carried out once in three repetitions, with five plants each. Data are presented as boxplots (*n* = 15). One way ANOVA followed by Duncan’s post hoc tests were used and different letters among these treatments indicate statistically significant differences (*p* < 0.05). HC: healthy control; DC: diseased control; RHF2.1–7: *B. marisflavi;* RHF4.1–25: *B. velezensis*; RHF4.1–26 and RHF3.1–20: *B. altitudinis*; BTH: S-methyl 1,2,3-benzothiadiazole-7-carbothioate. (**B**) Representative pictures of disease symptoms taken at the time of disease evaluation. Scale bar represents 1 cm.

A similar trend was observed when the *Bacillus* isolates were tested in an ASS. *B. velezensis* RHF4.1–25, and *B. altitudinis* RHF4.1–26 and RHF3.1–20 could significantly protect rice plants against *P. oryzae* in comparison to the diseased control. Among these strains, *B. velezensis* RHF4.1–25 and *B. altitudinis* RHF4.1–26 were as effective as the BTH treatment (Figure 2). In contrast, *B. marisflavi* RHF2.1–7 could not trigger ISR against rice blast. Furthermore, all tested isolates colonized rice roots ranging from 6.56 until 7.54 log CFU g^−1^ fresh roots (Table 3).

### 3.2. CLiP Production in B. altitudinis, B. velezensis and Mutants

**Figure 3:**
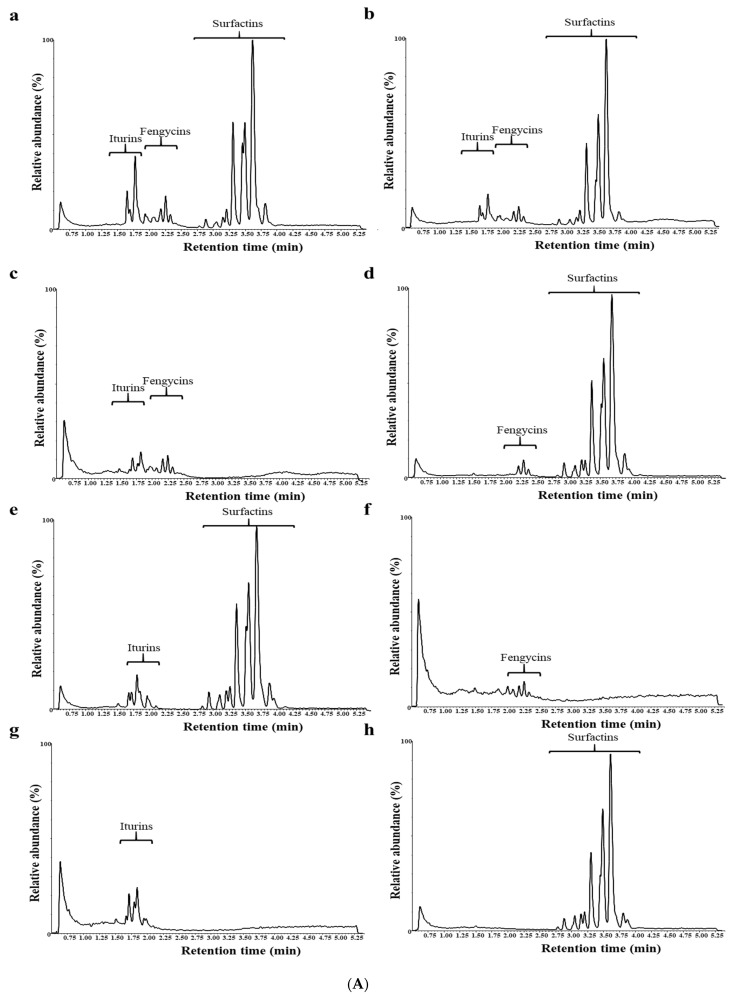

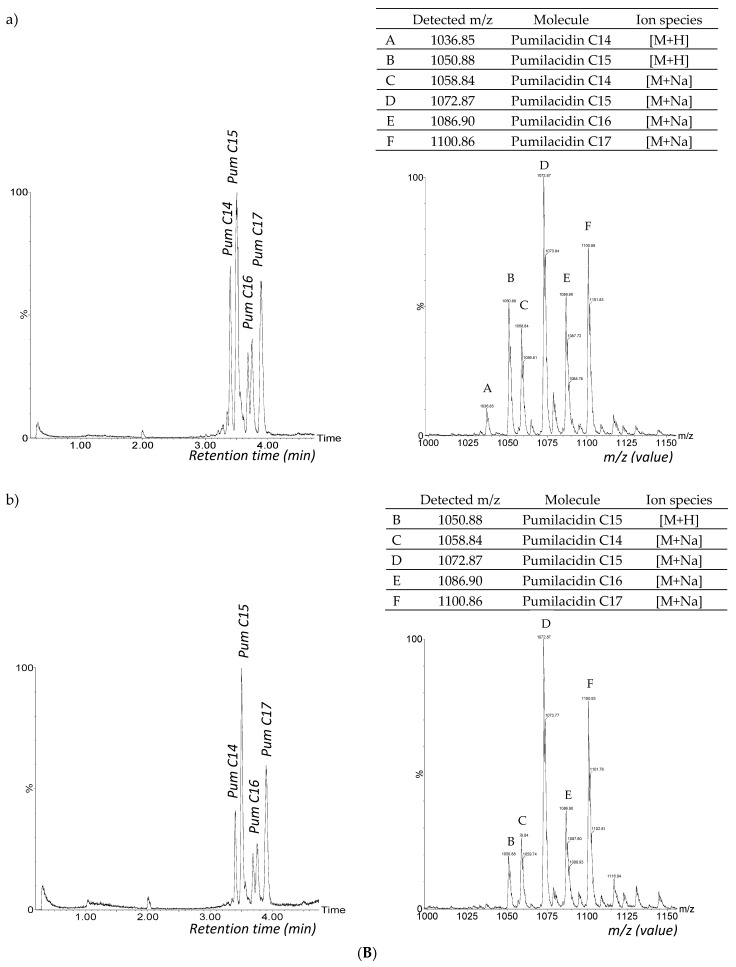
(**A**) Comparative LC–ESI–MS chromatograms of cell-free culture supernatant of (**a**) *B. velezensis* RHF4.1–25; (**b**) GA1: wild type of *B. velezensis*; (**c**) ∆*sfra*: surfactin mutant; (**d**) ∆*ituA*: iturin mutant; (**e**) ∆*fenA*: fengycin mutant; (**f**) ∆*sfra-ituA*: double mutant impaired in surfactin and iturin production; (**g**) ∆*sfrA-fenA*: double mutant impaired in surfactin and fengycin production; (**h**) ∆*fenA-ituA*: double mutant impaired in iturin and fengycin production after 24 h incubation. (**B**) LC–ESI–MS chromatograms of cell-free culture supernatant of the *B. altitudinis* strains RHF3.1–20 (**a**) and RHF4.1–26 (**b**). Various isoforms of pumilacidins (Pum) were detected, including Pum C14, Pum C15, Pum C16, and Pum C17 after 24 h incubation with the m/z values displayed in corresponding tables.

UPLC–MS analysis was performed to detect and quantify CLiPs produced by *B. velezensis* RHF4.1–25 and its CLiP profile was compared with *B. velezensis* GA1 (Figure 3, Table A2) (see Appendix A). *B. velezensis* RHF4.1–25 produces surfactins, fengycins and iturins, similar to the CLiPs produced by *B. velezensis* GA1. The CLiP profile of the various GA1 mutants including single and double CLiP mutants is also depicted in Figure 3 and Table A2. Both *B. altitudinis* strains RHF4.1–26 and RHF3.1–20 produce pumilacidins, as shown in Figure 3B.

### 3.3. Role of Cyclic Lipopeptides Produced by B. velezensis in ISR against P. oryzae VT5M1 in Potting Soil

**Figure 4:**
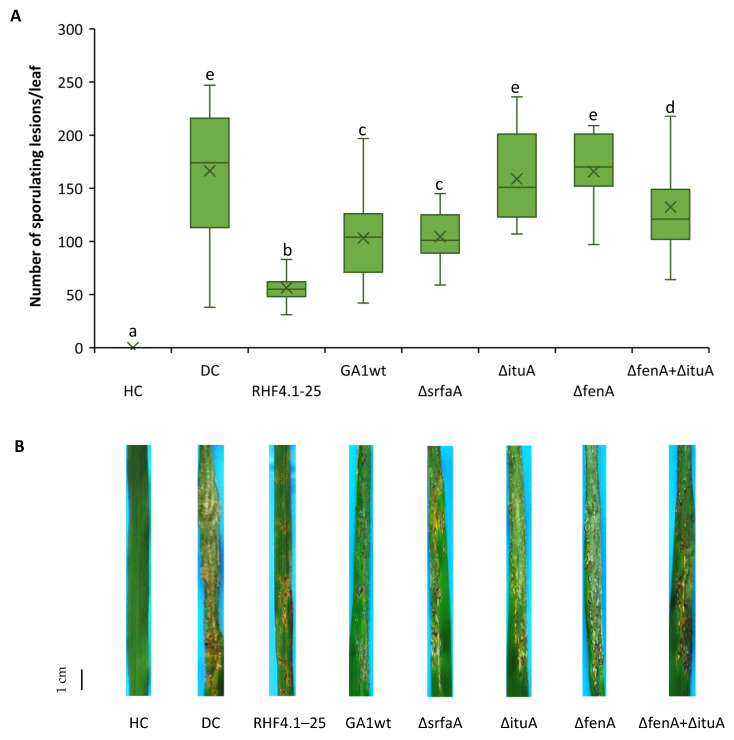
(**A**) Potential of *B. velezensis* isolates RHF4.1–25 and GA1 and GA1 mutants impaired in CLiP production to induce systemic resistance against *P. oryzae* in rice (variety CO39) grown in **potting soil**. Bacteria were applied by inoculating the roots of rice seedlings before transplanting and by a soil drench three days before pathogen inoculation. Four-week old rice plants were inoculated with 5 × 10^4^ spores mL^−1^ of *P. oryzae* VT5M1. Disease was evaluated six days post pathogen inoculation by counting the number of susceptible lesions. The experiment was conducted once in three repetitions with seven plants each. Data are presented as boxplots (*n* = 21). Univariate ANOVA followed by Duncan’s post hoc tests were used and different letters among these treatments indicate statistically significant differences (*p* < 0.05). RHF4.1–25 and GA1wt: wild type *B. velezensis*; ∆*srfaA*: surfactin mutant of GA1; ∆*ituA*: iturin mutant of GA1; ∆*fenA*: fengycin mutant of GA1; ∆*fenA* + ∆*ituA*: combined inoculation with fengycin and iturin mutant of GA1. (**B**) Representative pictures of disease symptoms taken at the time of disease evaluation. Scale bar represents 1 cm.

Since *B. velezensis* RHF4.1–25, an indigenous isolate obtained from rice roots in Vietnam, was the most effective in triggering ISR in previous experiments, its effect was compared with the closely related isolate *B. velezensis* GA1, a well-studied biocontrol agent that produces the CLiPs surfactin, iturin and fengycin. To study the role of CLiPs in ISR, in a first experiment single mutants of GA1 impaired in surfactin, iturin or fengycin were included in the assay. Figure 4 shows that *B. velezensis* RHF4.1–25, *B. velezensis* GA1 wild type (GA1wt) and its surfactin mutant (∆*srfaA)* could trigger the ISR against rice blast caused by *P. oryzae* VT5M1. These treatments could significantly protect rice plants in comparison with the diseased control (DC), corresponding to approximately 30%, 54% and 46% relative infection, respectively (Figures S1 and S2). In contrast, iturin (∆*ituA*) and fengycin (∆*fenA)*, mutants of *B. velezensis* GA1, lost the ability to trigger ISR, showing that both fengycin and iturin are needed to trigger ISR against *P. oryzae* VT5M1. The ISR capacity could be partially restored when both mutants were applied together (∆*ituA* + ∆*fenA).* It should be noted that in this experiment, *B. velezensis* RHF4.1–25 was more effective than *B. velezensis* GA1 (Figure 4).

In this experiment the root colonization of the tested *Bacillus* was in the range of 10^6^ CFU g^−1^ fresh root (Table 4). Root colonization of the CLiP mutants was not impaired in comparison with the wild type strain GA1.

**Figure 5:**
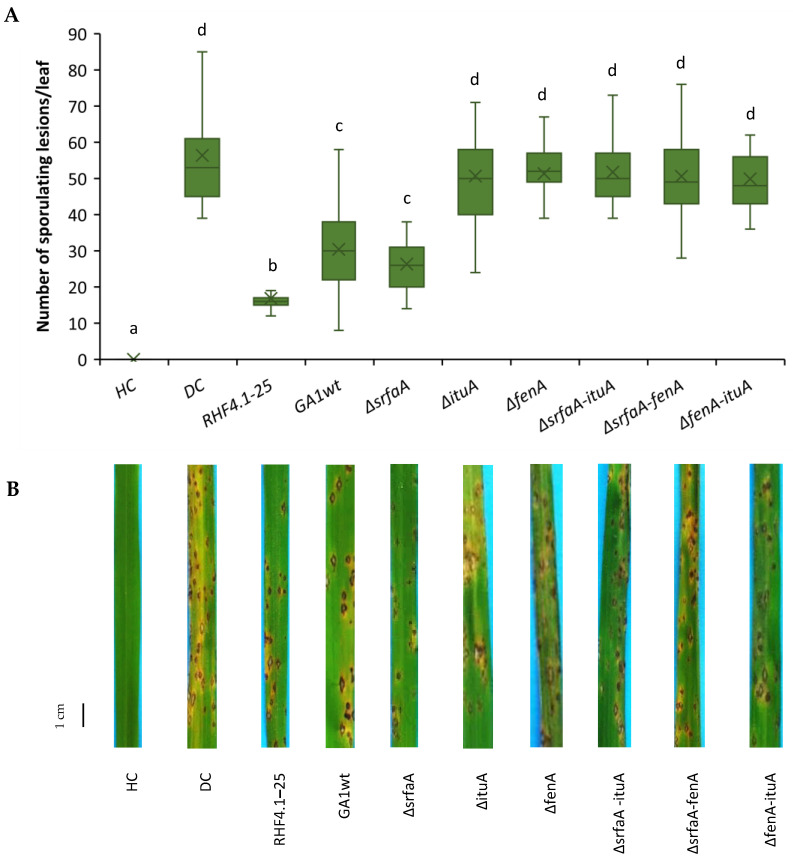
(**A**) Potential of *B. velezensis* isolates RHF4.1–25 and GA1, and GA1 mutants impaired in CLiP production to induce systemic resistance against *P. oryzae* in rice (variety CO39) grown in **potting soil**. Bacteria were applied by inoculating the roots of rice seedlings before transplanting and by a soil drench three days before pathogen inoculation. Four-week old rice plants were inoculated with 5 × 10^4^ spores mL^−1^ of *P. oryzae* VT5M1. Disease was evaluated six days post pathogen inoculation by counting the number of susceptible lesions. The experiment was conducted once in three repetitions, with seven plants each. Data are presented as boxplots (*n* = 21). Univariate ANOVA followed by Duncan’s post hoc tests were used and different letters among these treatments indicate statistically significant differences (*p* < 0.05). HC: healthy control; DC: diseased control: RHF4.1–25 and GA1wt: wild type *B. velezensis*; ∆*srfaA*: surfactin mutant; ∆*ituA*: iturin mutant; ∆*fenA*: fengycin mutant. ∆*srfaA-ituA*: double mutant impaired in surfactin and iturin production; ∆*srfaA-fenA*: double mutant impaired in surfactin and fengycin production; ∆*fenA-ituA*: double mutant impaired in iturin and fengycin production. (**B**) Representative pictures of disease symptoms taken at the time of disease evaluation. Scale bar represents 1 cm.

In a second experiment, double mutants of *B. velezensis* that are impaired in the production of two of the three CLiPs were also included (Figure 5).

All double mutants lost their ability to trigger ISR against rice blast, while the wild type strain *B. velezensis* GA1 and the surfactin mutant Δ*srfaA* were equally effective. In this experiment, *B. velezensis* RHF4.1–25 was significantly more effective than *B. velezensis* GA1. Root colonization of all isolates was in the range of 10^6^ CFU g^−1^ of fresh root (Table 5).

### 3.4. Role of Cyclic Lipopeptides in ISR of B. velezensis against P. oryzae VT5M1 in Acid Sulfate Soil

**Figure 6:**
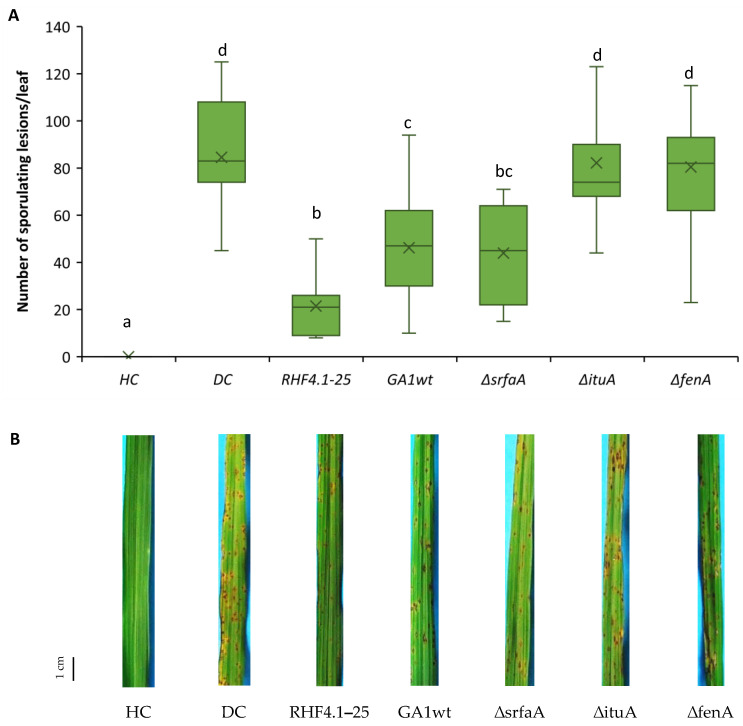
(**A**) Potential of *B. velezensis* isolates RHF4.1–25 and GA1, and GA1 mutants impaired in CLiP production to induce systemic resistance against *P. oryzae* in rice (variety Jasmine 85) grown in **acid sulfate soil**. Bacteria were applied by inoculating the roots of rice seedlings before transplanting and by a soil drench three days before pathogen inoculation. Four-week old rice plants were inoculated with 5 × 10^4^ spores mL^−1^ of *P. oryzae* VT5M1. Disease was evaluated six days post-pathogen inoculation by counting the number of susceptible lesions. The experiment was performed once in three repetitions with five plants each. Data are presented as boxplots (*n* = 15). Univariate ANOVA followed by Duncan’s post hoc tests were used and different letters among these treatments indicate statistically significant differences (*p* < 0.05). HC: healthy control; DC: diseased control: RHF4.1–25 and GA1wt: wild type *B. velezensis*; ∆*srfaA*: surfactin mutant of GA1; ∆*ituA*: iturin mutant of GA1; ∆*fenA*: fengycin mutant of GA1. (**B**) Representative pictures of disease symptoms taken at the time of disease evaluation. Scale bar represents 1 cm.

With regard to ISR against rice blast under ASS conditions, *Bacillus* strains including RHF4.1–25, GA1wt and only GA1 single mutants were chosen to investigate the capacity of these strains to protect the rice plants against *P. oryzae* VT5M1 due to the limited availability of Vietnamese ASSs to set up experiments and the consistent results above in potting soils. The results show that the ISR capacity of those isolates which could effectively protect rice plants in potting soils, worked consistently under ASS conditions. Specifically, *B. velezensis* RHF4.1–25, *B. velezensis* GA1wt and Δ*srfaA* isolates could significantly protect the rice plants against *P. oryzae* compared to the diseased control. Among these strains, RHF4.1–25 was again most effective, but not significantly different from the Δ*srfaA* mutant (Figure 6). In contrast, mutants unable to produce iturin or fengycin could not significantly trigger ISR in rice blast and reduced disease severity only by 5% (Figure S3). Furthermore, all tested isolates colonized rice roots in the range of 10^6^ CFU g^−1^ fresh roots (Table 6).

### 3.5. In Vitro Antagonism of P. oryzae Using Cell-Free Supernatants of B. velezensis Wild Type Strains and CLiP Mutants

**Figure 7:**
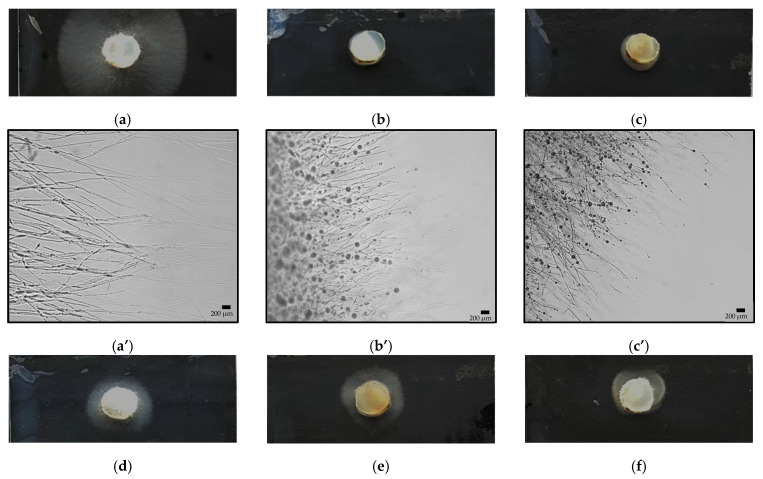

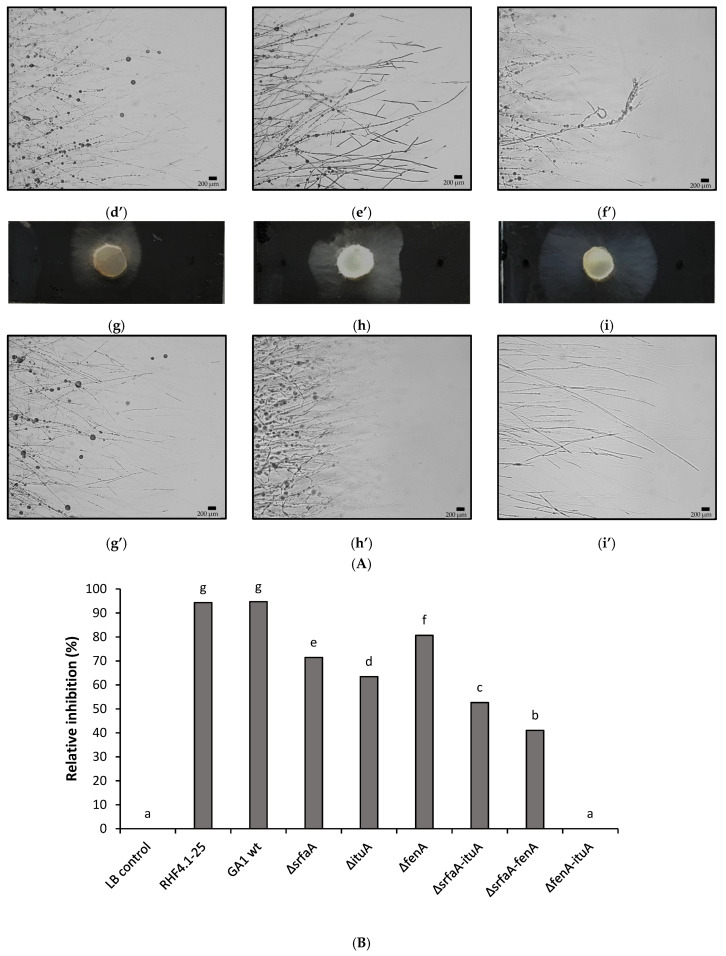
(**A**) Microscopic assay showing the effect of cell-free supernatants obtained from CLiPs-producing *B. velezensis* RHF4.1–25, *B. velezensis* GA1wt and its mutants on mycelial growth of *P. oryzae* VT5M1. Treatments include (**a**,**a’**) control (LB broth); (**b**,**b’**) *B. velezensis* RHF4.1–25; (**c**,**c’**) *B. velezensis* GA1 wild type (GA1wt), (**d**,**d’**) *B. velezensis* GA1*-*∆*srfaA*; (**e**,**e’**) *B. velezensis* GA1*-*∆*ituA*; (**f**,**f’**) *B. velezensis* GA1*-*∆*fenA*; (**g**,**g’**) *B. velezensis* GA1*-*∆*srfaA-ituA*; (**h**,**h’**) *B. velezensis* GA1*-*∆*srfaA-fenA*; (**i**,**i’**) *B. velezensis* GA1*-*∆*fenA-ituA*. (**B**) Direct antagonistic effects of cell-free supernatants obtained from CLiPs-producing *B. velezensis* RHF4.1–25, *B. velezensis* GA1wt and GA1 mutants against *P. oryzae* VT5M1. Results are expressed as relative inhibition in comparison with the control. ∆*srfaA*: surfactin mutant; ∆*ituA*: iturin mutant; ∆*fenA*: fengycin mutant. ∆*srfaA-ituA*: double mutant impaired in surfactin and iturin production; ∆*srfaA-fenA*: double mutant impaired in surfactin and fengycin production; ∆*fenA-ituA*: double mutant impaired in iturin and fengycin production. Each treatment was repeated five times and the experiment was done twice. Univariate ANOVA followed by Duncan’s post hoc tests were used and different letters among these treatments indicate statistically significant differences (*p* < 0.05).

Microscopic assays were conducted to investigate direct effects of CLiP containing supernatants on the mycelial growth of *P. oryzae* VT5M1. Figure 7 shows that application of cell-free supernatants obtained from the wild type *B. velezensis* strains RHF4.1–25 and GA1 strongly suppressed the growth of *P. oryzae* VT5M1 with more than 94%. More importantly, abnormal hyphal fragments were also formed by those treatments (Figure 7A), as compared with that of the LB control. Supernatants containing two CLiPs (obtained from ∆*srfaA*, ∆*ituA* and ∆*fenA*) also strongly inhibited the growth of *P. oryzae* VT5M1 (from 63% to 80%), supernatants that contained both surfactin and iturin gave the best results (about 80% inhibition). Additionally, supernatants that only contained fengycin or iturin gave an intermediate inhibition from 41% to 52%. No inhibition zone was formed between fungus and supernatant that only contained surfactin (Figure 7B).

### 3.6. Influence of Cell-Free Culture Filtrates on P. oryzae Spore Germination and Appressoria Formation

**Figure 8:**
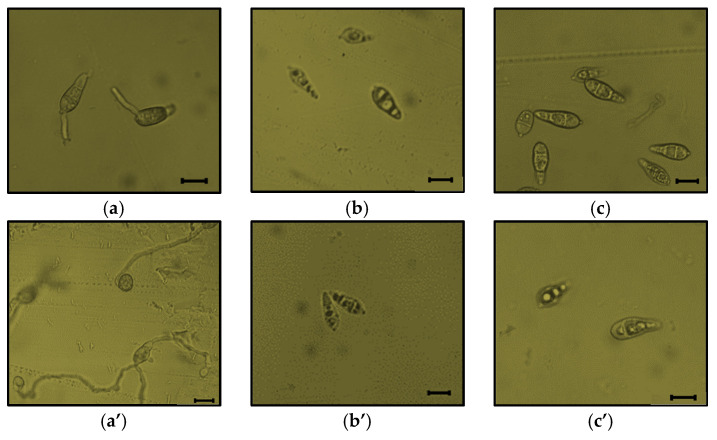

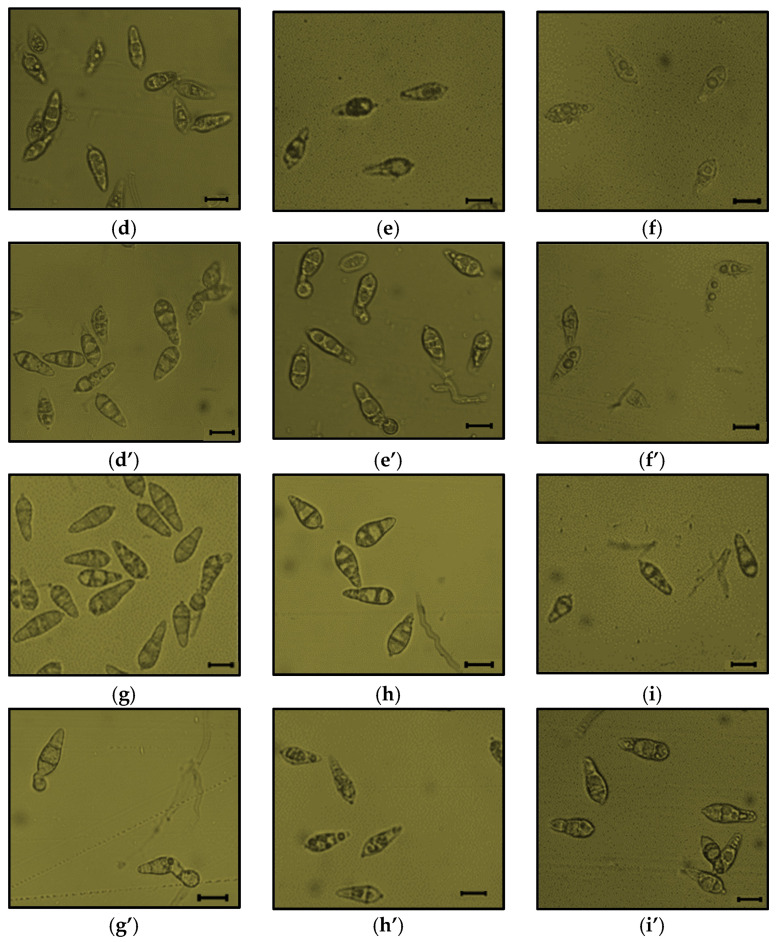
Effect of cell-free supernatants obtained from CLiPs-producing *B. velezensis* RHF4.1–25, *B. velezensis* GA1wt and its mutants on spore germination (at four hours post incubation) and appressorium formation (at eight hours post incubation) of *P. oryzae* VT5M1. Treatments include: (**a**) (4 hpi) and (**a’**) (8 hpi), control (LB broth); (**b**) (4 hpi) and (**b’**) (8hpi), RHF4.1–25; (**c**) (4 hpi) and (**c’**) (8 hpi); GA1wt, (**d**) (4 hpi) and (**d’**) (8 hpi); GA1∆*srfaA*; (**e**) (4 hpi) and (**e’**) (8 hpi), GA1∆*ituA*; (**f**) (4 hpi) and (**f’**) (8 hpi), GA1∆*fenA*; (**g**) (4 hpi) and (**g’**) (8 hpi), GA1∆*srfaA-ituA*; (**h**) (4 hpi) and (**h’**) (8 hpi), GA1∆*srfaA-fenA*; (**i**) (4 hpi) and (**i’**) (8 hpi), GA1∆*fenA-ituA*. Scale bar represents 100 µm.

This experiment was performed to study the role of CLiPs on *P oryzae* VT5M1 spore germination and appressoria formation. Following incubation in favorable environmental conditions for fungal growth, the percentage of conidial germination and appressoria formation in the LB control were 97% and 100%, respectively (Table 7). Interestingly, all cell-free culture filtrates obtained from wild type and mutant *B. velezensis* strains effectively inhibited conidial germination. More so, appressorium formation was not recorded since there was no spore germination in inoculated treatments. In addition to this, the CLiPs-containing supernatants damaged the spores and caused malformation of germ tubes (Figure 8).

### 3.7. Direct Effect of Cell-Free Supernatants to Reduce Rice Blast Symptoms Caused by P. oryzae Spores

**Figure 9:**
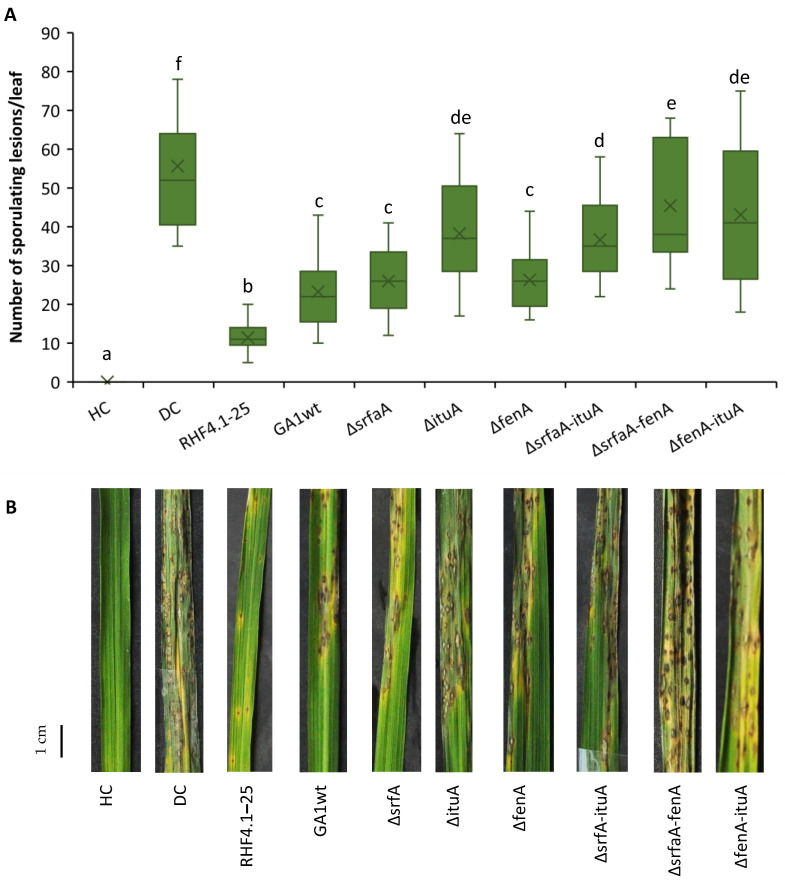
(**A**) Direct antagonistic activity of cell-free supernatants obtained from CLiPs-producing *B. velezensis* RHF4.1–25, *B. velezensis* GA1 wild type and its mutants against *P. oryzae* VT5M1. Cell-free supernatants of the different bacterial isolates were mixed with a spore suspension of *P. oryzae* VT5M1 and sprayed on 4–week old rice plants grown in potting soil. Disease was evaluated six days post pathogen inoculation by counting the number of susceptible lesions. The experiment was performed once in three repetitions, with seven plants each. Data are presented as boxplots (*n* = 21). Univariate ANOVA followed by Duncan’s post hoc tests were used and different letters among these treatments indicate statistically significant differences (*p* < 0.05). HC: healthy control; DC: diseased control: RHF4.1–25 and GA1wt: wild type *B. velezensis*; ∆*srfaA*: surfactin mutant; ∆*ituA*: iturin mutant; ∆*fenA*: fengycin mutant. ∆*srfaA-ituA*: double mutant impaired in surfactin and iturin production; ∆*srfaA-fenA*: double mutant impaired in surfactin and fengycin production; ∆*fenA-ituA*: double mutant impaired in iturin and fengycin production. (**B**) Representative pictures of disease symptoms taken at the time of disease evaluation. Scale bar represents 1 cm.

Since significantly effective inhibition of CLiPs-producing *Bacillus* isolates on mycelial growth as well as on spore germination of *P. oryzae* VT5M1 was shown in experiments above, this assay was carried out to evaluate the capacity of the CLiPs in disease severity reduction by *in planta* direct antagonism. Based on the results of this plant experiment (Figure 9), application of all CLiPs-producing *Bacillus* isolates could significantly reduce lesion numbers compared to that of the untreated control (DC, disease control).

Furthermore, there were remarkable differences in disease symptom reduction between these treatments caused by spraying a 50% (*v*/*v*) concentration of mixed cell-free supernatants and spores. Interestingly, in comparison with *B. velezensis* GA1wt and its mutants, cell-free culture filtrates from *B. velezensis* RHF4.1–25 were more effective in disease suppression (Figure S4). Among GA1wt strain and its mutants, the supernatants of GA1wt, ∆*srfaA* and ∆*fenA* provided a significant reduction in lesion numbers, as well as relative infection, compared to that of the remaining GA1 mutants. Along with supernatant-producing ∆*ituA*, double mutants, namely, ∆*srfaA*-*ituA*, ∆*srfaA-fenA* and ∆*fenA-ituA* were less effective than other tested GA1 strains, but could decrease disease symptoms by 20% to 35% compared to the diseased control.

## 4. Discussion

In this study, we screened the capacity of four representative *Bacillus* isolates from ASSs in Vietnam to elicit ISR against rice blast disease and selected potential biocontrol agents for further studies. *B. marisflavi* could not successfully induce resistance to rice blast, in contrast, *B. altitudinis* and *B. velezensis* strains could effectively protect the rice plants against blast disease by inducing resistance upon root inoculation. These strains consistently triggered resistance to the blast disease in rice grown in both potting and acid sulfate soil conditions. *B. velezensis* RHF4.1–25 was the most successful in triggering ISR against blast disease. Our study is the first report on the capacity of indigenous *Bacillus* isolates to trigger ISR against blast disease on rice grown in ASSs. 

The two used *B. altitudinis* strains could protect rice against blast disease by triggering ISR. *B. altitudinis* has been implicated in biocontrol in rice against the sheath blight pathogen *Rhizoctonia solani* by ISR [40] and against the bacterial blight pathogen *Xanthomonas oryzae* pv*. oryzae* via direct effects [41]. Both strains produce the pumilacidin-type of CLiPs. Pumilacidins are members of the surfactin family. The compounds differ from surfactin with a leucine in position 4 instead of a valine and an isoleucine or valine at position 7 instead of a leucine [42]. Pumilacidins are known to be produced by *B. pumilus* [43,44] and by *B. safensis* [45], two species closely related to *B. altitudinis.* To our knowledge, there are no studies showing that *B. altitudinis* strains produce pumilacidins or that they are active against rice blast. In this context, it should be noticed we could not demonstrate a role for surfactin produced by *B. velezensis* GA1 in triggering ISR against *P. oryzae*. However, variations in molecular structure of the peptide part can impact the physicochemical properties of a CLiP. The presence of a Leu4 in pumilacidin instead of a Val4, as in surfactin, appears to increase the critical micellar concentration value [42]. The final surface tension value of pumilacidin is also higher when compared to surfactin [45], so it cannot be excluded that this has an effect on ISR in rice. It remains to be investigated whether pumilacidins do play a role in the observed ISR against *P. oryzae*. 

The ISR determinants of *B. velezensis* were deciphered in this study. Our studies have revealed that upon root inoculation, surfactin-, fengycin- and iturin-type CLiPs-producing RHF4.1–25 and *B.* velezensis GA1 could effectively protect rice plants against blast disease by triggering ISR. Furthermore, to shed more light on the ISR capacity of these CLiPs, single (∆*srfaA*, ∆*ituA* and ∆*fenA*) and double mutants (∆*srfaA*-*ituA*, ∆*srfaA-fenA* and ∆*fenA-ituA*) of GA1 were tested in this study to investigate their ISR capacity against *P. oryzae* VT5M1 in rice. Both fengycin and iturin are needed to trigger ISR against rice blast disease, since mutants impaired in fengycin or iturin or in both fengycin and iturin production could no longer protect the plants against rice blast disease. Additionally, mutant strains that only produce a single CLiP could not successfully protect plant against *P. oryzae* VT5M1 by triggering ISR. These data suggest that fengycin and iturin act synergistically to cause the ISR response, while surfactin appears not to play a role in ISR. This is in contrast with a previous study in which surfactin triggered ISR against *P. oryzae* in perennial ryegrass [46], indicating that the monocots rice and ryegrass do not react to lipopeptides in a similar way. Our findings are in agreement with a previous study in which surfactin and fengycin could elicit resistance against sheath blight caused by *Rhizoctonia solani* in rice*,* but were not effective against *P. oryzae* [31]. Another recent study showed that *B. subtilis* strain BJ–1, a strain isolated from a contaminated *P. oryzae* culture plate, could trigger ISR against rice blast on rice by seed inoculation, however, the study did not determine the effect of individual lipopeptides [15]. In many previous studies, the role of surfactin, iturin, or surfactin and fengycin together in triggering ISR in various pathosystems could be demonstrated. It has been reported that surfactin and fengycin produced by *B. subtilis* strain S499 could protect bean plants against *Botrytis cinerea* by triggering ISR [30]. Furthermore, iturin family CLiPs such as mycosubtilin and bacillomycin have also consistently triggered ISR activity against phytopathogens in various host plants [47]. These results clearly show that the capacity of CLiPs to induce resistance is pathogen and plant dependent. In addition to *Bacillus*, CLiP-producing *Pseudomonas* strains have also successfully been used to control rice blast by ISR. The capacity of the ISR-inducing *Pseudomonas* strains COR10, COW10 and COR5 to protect rice plants against *P. oryzae* was demonstrated to be due to the production of the CLiPs lokisin, WLIP and entolysin, respectively [25]. 

Beside indirect antagonism by inducing resistance, direct effects of cell-free supernatants produced by *B. velezensis* on mycelial growth, spore germination and appressorium formation of *P. oryzae* were also investigated. Our in vitro tests suggest that iturin and fengycin are effective in suppressing both the mycelial growth and spore germination of *P. oryzae,* whereas surfactin only affects spore germination. It has been shown before that surfactin produced by *B. licheniformis* BC98 could suppress the spore germination of *P. oryzae* B157 at a concentration of 1 µg mL^−1^ [48]. Iturin appears to be the most effective at inhibiting mycelial growth since the mutant impaired in fengycin production still showed a high relative inhibition. Surfactin-, fengycin- and iturin-producing *Bacillus* spp. are well-known for their direct antagonism against diverse phytopathogens in different host plants [33,47,49]. Surfactins are powerful biosurfactants that interact with lipid bilayers, and disrupt and solubilize lipid bilayers at high concentrations [33]. Fengycins and iturins directly affect fungal cell membranes and finally cause cell death [33,43,50]. Our study suggests that the presence of all three CLiPs types resulted in the best antagonistic effects on the growth of *P. oryzae* VT5M1. Interestingly, supernatants containing these CLiPs also completely suppressed spore germination of the fungus *P. oryzae* VT5M1. This result is consistent with earlier reports about the influence of surfactin- and fengycin-type CLiPs on conidial germination and appressorium formation in *P. oryzae* [15]. Besides, we tested the mixture of bacterial cell-free supernatants and the spore solutions as a spray on rice plants. In this case the supernatant that only contains surfactin could also significantly reduce disease severity compared to the diseased control treatment, probably because the compound could inhibit spore germination. Moreover, our results also show that the combination of surfactin-, fengycin-, iturin-type CLiPs resulted in the best protective effect on rice plants. 

Interestingly, in comparison with *B. velezensis* GA1wt, *B. velezensis* RHF4.1–25 obtained from ASS could protect rice plants more effectively in both direct and indirect antagonism against blast disease caused by *P. oryzae* VT5M1. This is not due to a possibly better adaptation to ASS, since the difference in effectiveness was also observed in potting soil and in direct antagonism. Both strains also showed a very comparable root colonization in all plant assays. Differences in effectiveness may be due to differences in the regulation of CLiP production or in CLiP ratio. We are currently performing an in-depth genome analysis of both strains to find out why *B. velezensis* strain RHF4.1–25 performs better than *B. velezensis* GA1 in *P. oryzae* control. 

## 5. Conclusions

Our study highlights that indigenous *B. altitudinis* and *B. velezensis* strains isolated from rice rhizosphere in ASSs in Vietnam could trigger ISR against rice blast disease. Furthermore, this study has elucidated the role of surfactin, fengycin, and iturin in controlling rice blast disease by ISR and direct antagonism. Fengycin and iturin are both needed to elicit ISR against blast disease, suggesting a synergistic interaction. Apart from ISR, our results also highlight the important role of surfactin, fengycin and iturin in the direct inhibition of *P. oryzae*. Both fengycin and iturin inhibit spore germination and mycelial growth, while surfactin only inhibits spore germination. This work also demonstrates that *B. velezensis* RHF4.1–25 isolated from rice rhizosphere in Vietnamese ASSs is more effective than *B. velezensis* GA1 to control rice blast by both ISR and direct antagonism, although both strains produce the same CLiPs. It is worthwhile to further explore the potential of *B. velezensis* strain RHF4.1–25 as biocontrol agent against rice blast in field conditions in Vietnam by applying the strain via seed application, root inoculation and/or spraying on rice leaves. 

## Figures and Tables

**Table 1:** Microorganisms used in this study and their relevant characteristics.

Strain	Relevant Characteristics	References
*Bacillus altitudinis* group		
RHF4.1–26	Pumilacidin-producer from rice rhizosphere grown in ASS, Vietnam	This study
RHF3.1–20	Pumilacidin-producer from rice rhizosphere grown in ASS, Vietnam	This study
*Bacillus marisflavi* group		
RHF2.1–7	Non-CLiP-producer from rice rhizosphere grown in ASS, Vietnam	This study
*Bacillus velezensis* group		
RHF4.1–25	Surfactin, iturin and fengycin-producer from rice rhizosphere grown in ASS, Vietnam	This study
GA1wt	Wild type: srf^+^, itu^+^, fen^+^	[35]
GA1∆*srfaA*	Surfactin mutant: srf^−^, itu^+^, fen^+^	[37]
GA1∆*ituA*	Iturin mutant: itu^−^, srf^+^, fen^+^	[37]
GA1∆*fenA*	Fengycin mutant: fen^−^, itu^+^, srf^+^,	[37]
GA1∆*srfaA-ituA*	Surfactin and iturin mutant: srf^−^, itu^−^, fen^+^	This study
GA1∆*srfaA-fenA*	Surfactin and fengycin mutant: srf^−^, fen^−^, itu^+^	This study
GA1∆*fenA-ituA*	Iturin and fengycin mutant: itu^−^, fen^−^, srf^+^	This study
*Pyricularia oryzae* VT5M1	Rice blast pathogen from Vietnam	[12]

Abbreviations: ASS: acid sulfate soil; Srf: surfactin; Itu: iturin; Fen: Fengycin.

**Table 2:** Population of *Bacillus* isolates applied in ISR assay on rice roots grown in potting soil (Figure 1).

Treatment	Population Density (log CFU g^−1^ of Fresh Root)
*B. marisflavi* RHF2.1–7	8.41 ± 0.06 ^c^
*B. velezensis* RHF4.1–25	7.97 ± 0.11 ^b^
*B. altitudinis* RHF4.1–26	7.70 ± 0.08 ^a^
*B. altitudinis* RHF3.1–20	8.15 ± 0.32 ^b^

No used *Bacillus* bacteria were found in noninoculated treatments. Means and standard deviation are presented. Values followed by distinct letters are significantly different (Duncan’s test, *p* < 0.05).

**Table 3:** Population of *Bacillus i*solates applied in ISR assay on rice roots grown in acid sulfate soil (Figure 2).

Treatment	Population Density (log CFU g^−1^ of Fresh Root)
*B. marisflavi* RHF2.1–7	7.31 ± 0.59 ^b^
*B. velezensis* RHF4.1–25	6.56 ± 0.59 ^a^
*B. altitudinis* RHF4.1–26	7.41 ± 0.65 ^b^
*B. altitudinis* RHF3.1–20	7.54 ± 0.37 ^b^

No used *Bacillus* bacteria were found in noninoculated treatments. Means and standard deviation are presented. Values followed by distinct letters are significantly different (Duncan’s test, *p* < 0.05).

**Table 4:** Population of *Bacillus* isolates applied in ISR assay on rice roots grown in potting soils (Figure 4).

Treatment	CLPs Produced	Population Density (in log CFU g^−1^ of Fresh Root)
*B. velezensis* RHF4.1–25	Surfactin, iturin and fengycin	6.16 ± 0.63 ^a^
*B. velezensis* GA1wt	Surfactin, iturin and fengycin	6.12 ± 0.57 ^a^
*B. velezensis* GA1∆*srfaA*	Iturin and fengycin	6.60 ± 0.31 ^a^
*B. velezensis* GA1∆*ituA*	Surfactin and fengycin	6.41 ± 0.52 ^a^
*B. velezensis* GA1∆*fenA*	Surfactin and iturin	6.38 ± 0.50 ^a^
*B. velezensis* GA1∆*fenA-ituA*	Surfactin, iturin and fengycin	6.26 ± 0.40 ^a^

No used *Bacillus* bacteria were found in noninoculated treatments. Means and standard deviation are presented. Values followed by distinct letters are significantly different (Duncan’s test, *p* < 0.05).

**Table 5:** Population of *Bacillus* isolates applied in ISR assay on rice roots grown in potting soils (Figure 5).

Treatment	CLPs Produced	Population Density (in log CFU g^−1^ of Fresh Root)
*B. velezensis* RHF4.1–25	Surfactin, iturin and fengycin	6.32 ± 0.42 ^a^
*B. velezensis* GA1wt	Surfactin, iturin and fengycin	6.49 ± 0.23 ^a^
*B. velezensis* GA1∆*srfaA*	Iturin and fengycin	6.46 ± 0.45 ^a^
*B. velezensis* GA1∆*ituA*	Surfactin and fengycin	6.52 ± 0.30 ^a^
*B. velezensis* GA1∆*fenA*	Surfactin and iturin	6.38 ± 0.29 ^a^
*B. velezensis* GA1∆*srfaA-ituA*	Fengycin	6.54 ± 0.28 ^a^
*B. velezensis* GA1∆*srfaA-fenA*	Iturin	6.45 ± 0.30 ^a^
*B. velezensis* GA1∆*fenA-ituA*	Surfactin	6.70 ± 0.64 ^a^

No used *Bacillus* bacteria were found in noninoculated treatments. Means and standard deviation are presented. Values followed by distinct letters are significantly different (Duncan’s test*, p* < 0.05)

**Table 6:** Population of *Bacillus* isolates applied in ISR assay on rice roots grown in acid sulfhate soils (Figure 6).

Treatment	CLPs Produced	Population Density (in log CFU g^−1^ of Fresh Root)
*B. velezensis* RHF4.1–25	Surfactin, iturin and fengycin	6.77 ± 0.31 ^a^
*B. velezensis* GA1wt	Surfactin, iturin and fengycin	6.66 ± 0.40 ^a^
*B. velezensis* GA1∆*srfaA*	Iturin and fengycin	6.21 ± 0.13 ^a^
*B. velezensis* GA1∆*ItuA*	Surfactin and fengycin	6.33 ± 0.16 ^a^
*B. velezensis* GA1∆*fenA*	Surfactin and iturin	6.50 ± 0.17 ^a^

No used *Bacillus* bacteria were found in noninoculated treatments. Means and standard deviation are presented. Values followed by distinct letters are significantly different (Duncan’s test, *p* < 0.05).

**Table 7:** Effects of cell-free culture supernatant obtained from *B. velezensis* RHF4.1–25, *B. velezensis* GA1 or its CLiP mutants on spore germination of *P. oryzae* VT5M1 (Figure 8).

Treatment	CLPs Produced	Spore Germination (%)
Control (LB broth)	-	97 ± 1
*B. velezensis* RHF4.1–25	Surfactin, iturin and fengycin	0 ± 0
*B. velezensis* GA1wt	Surfactin, iturin and fengycin	0 ± 0
*B. velezensis* GA1∆*srfaA*	Iturin and fengycin	0 ± 0
*B. velezensis* GA1∆*ituA*	Surfactin and fengycin	0 ± 0
*B. velezensis* GA1∆*fenA*	Surfactin and iturin	0 ± 0
*B. velezensis* GA1∆*srfaA-ituA*	Fengycin	6 ± 3
*B. velezensis* GA1∆*srfaA-fenA*	Iturin	0 ± 0
*B. velezensis* GA1∆*fenA-ituA*	Surfactin	0 ± 0

Values indicate percentage of spore germination ± standard deviation (*n* = 50).

## Data Availability

Not applicable.

## Supplementary Materials

The following are available online at https://www.mdpi.com/article/10.3390/microorganisms9071441/s1, Figure S1: potential of *B. velezensis* isolates RHF4.1–25 and GA1 and GA1 mutants impaired in CLiP production to induce systemic resistance against *P. oryzae* in rice (variety CO39) grown in potting soil. Figure S2: potential of *B. velezensis* isolates RHF4.1–25 and GA1, and GA1 mutants impaired in CLiP production to induce systemic resistance against *P. oryzae* in rice (variety CO39) grown in potting soil. Figure S3: potential of *B. velezensis* isolates RHF4.1–25 and GA1, and GA1 mutants impaired in CLiP production to induce systemic resistance against *P. oryzae* in rice (variety Jasmine 85) grown in acid sulfate soil. Figure S4: direct antagonistic activity of cell-free supernatants obtained from CLiPs-producing *B. velezensis* RHF4.1–25, *B. velezensis* GA1 wild type and its mutants against *P. oryzae* VT5M1.

## Appendix A

**Table A1:** Primers used for the construction of *B. velezensis* GA1 mutants.

	Primer Name	Primer Sequence (5′->3′)	Targeted Genes	Reference
**Deletion Mutant**				
***B. velezensis*** **GA1**				
	UpsrfaAF	TCAGCAAAACTGCGTGGTAG	*srfaA*	[37]
	UpsrfaAR	CCAATTTTCGAATTCTTTTACCGCGATAAAAAGTTATTTCCATATGTGTGC		
	DwsrfAF	CAGCTCCAGATCCTCTACGCCGGACACGCTTTATATCGTGCCGAA		
	DwsrfAR	AAGAAATGATCATAAATACC		
	UpFenAF	AGCAAAAACCGGGTCACTAA	*fenA*	[37]
	UpFenAR	CCAATTTTCGAATTCTTTTACCGCGTTCGTCTGACATGACAAGCA		
	DwFenAF	CAGCTCCAGATCCTCTACGCCGGACAAAGGACTTTAATTTCATAAAAAGGTG		
	DwFenAR	CCTTTTTGAGAAGAGAAGAAAAAG		
	UpItuAF	ATGCAGGAAATAGGGGTGAA	*ituA*	[37]
	UpItuAR	CCAATTTTCGAATTCTTTTACCGCGGGTATACATAGGTCCCCTCCTG		
	DwItuAF	CAGCTCCAGATCCTCTACGCCGGACCAATTGAACTTTTAGGGAAAAGCA		
	DwItuAR	GCGACTAACGTATCGGGTTG		
**Antibiotic marker**				
	PhleoF	GTCATAGCTGTTTCCTGCCAAAAGGGGGTTTCATTTT	Phleomycin marker	[37]
	PhleoR	ACTGGCCGTCGTTTTACTCCAATAAATGCGACACCAA		

**Table A2:** Percentage of CLiPs produced by *Bacillus velezensis* RHF4.1–25 and mutants of *B. velezensis* GA1 in comparison with *B. velezensis* GA1 wild type.

Strain ^a^	CLPs Produced (%) ^b^		
	Surfactins	Fengycins	Iturins
*B. velezensis* GA1wt	100 ± 4.2	100 ± 2.2	100 ± 4.8
*B. velezensis* GA1∆*srfaA*	0 ± 0	32 ± 2.8	37 ± 4.3
*B. velezensis* GA1∆*ituA*	129 ± 1.3	82 ± 0.6	0 ± 0
*B. velezensis* GA1∆*fenA*	139 ± 1.3	0 ± 0	121 ± 0.9
*B. velezensis* GA1∆*srfaA-ituA*	0 ± 0	18 ± 9.7	0 ± 0
*B. velezensis* GA1∆*srfaA-fenA*	0 ± 0	0 ± 0	69 ± 2.1
*B. velezensis* GA1∆*fenA-ituA*	137 ± 2.9	0 ± 0	0 ± 0
*B. velezensis* RHF4.1–25	81 ± 15	104 ± 12.9	96 ± 18

^a^ ∆*srfaA*: surfactin mutant; ∆*ituA*: iturin mutant; ∆*fenA*: fengycin mutant. ∆*srfaA-ituA*: double mutant impaired in surfactin and iturin production; ∆*srfaA-fenA*: double mutant impaired in surfactin and fengycin production; ∆*fenA-ituA*: double mutant impaired in iturin and fengycin production. ^b^ Values indicate percentage of CLiP production in comparison with *B. velezensis* GA1wt ± standard deviation (*n* = 3).
