# Supplementary material for: Bacillus Cyclic Lipopeptides Iturin and Fengycin Control Rice Blast Caused by Pyricularia oryzae in Potting and Acid Sulfate Soils by Direct Antagonism and Induced Systemic Resistance
Source: Microorganisms. 2021 Jul 3;9(7):1441. doi: 10.3390/microorganisms9071441 (PMC8305041; doi:10.3390/microorganisms9071441)
Supplement: Supplementary file 1 [file microorganisms-09-01441-s001.zip › microorganisms-1282465-SI.pdf]

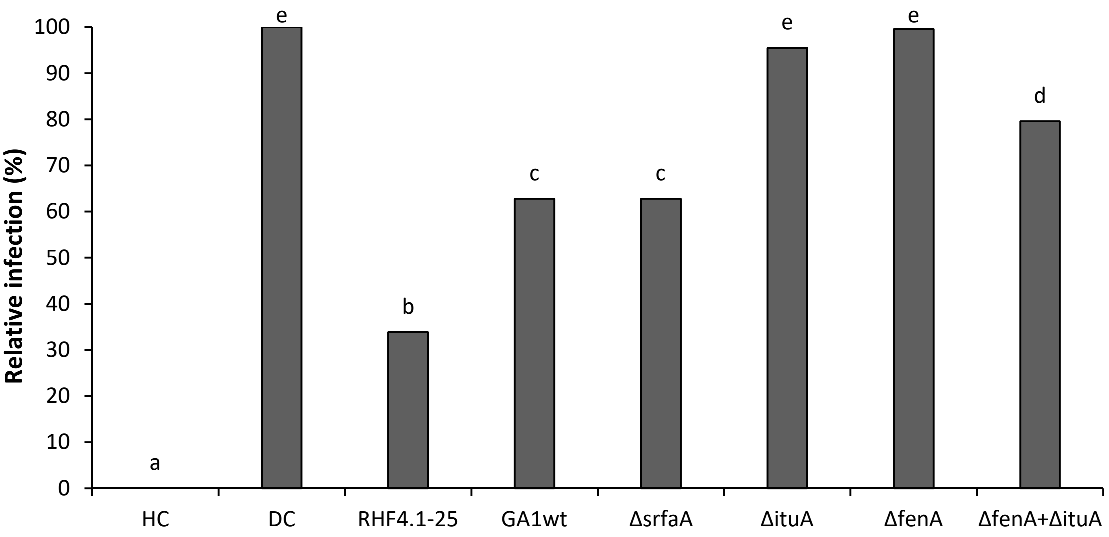


**Figure S1.** Potential of *B. velezensis* isolates RHF4.1-25 and GA1 and GA1 mutants impaired in CLiP production to induce systemic resistance against *P. oryzae* in rice (variety CO39) grown in sterile potting soil. Data from Figure 4 are here expressed as % relative infection in comparison with the diseased control (n=27). Univariate ANOVA followed by Duncan's post hoc tests were used and different letters among these treatments indicate statistically significant differences  (P < 0.05). HC: healthy control; DC: diseased control; RHF4.1-25 and GA1: wild type *B. velezensis*;  Δ*srfaA*: surfactin mutant of GA1; Δ*ituA*: iturin mutant of GA1; Δ*fenA*: fengycin mutant of GA1; Δ*fenA* + Δ*ituA*: combined inoculation with fengycin and iturin mutant of GA1.

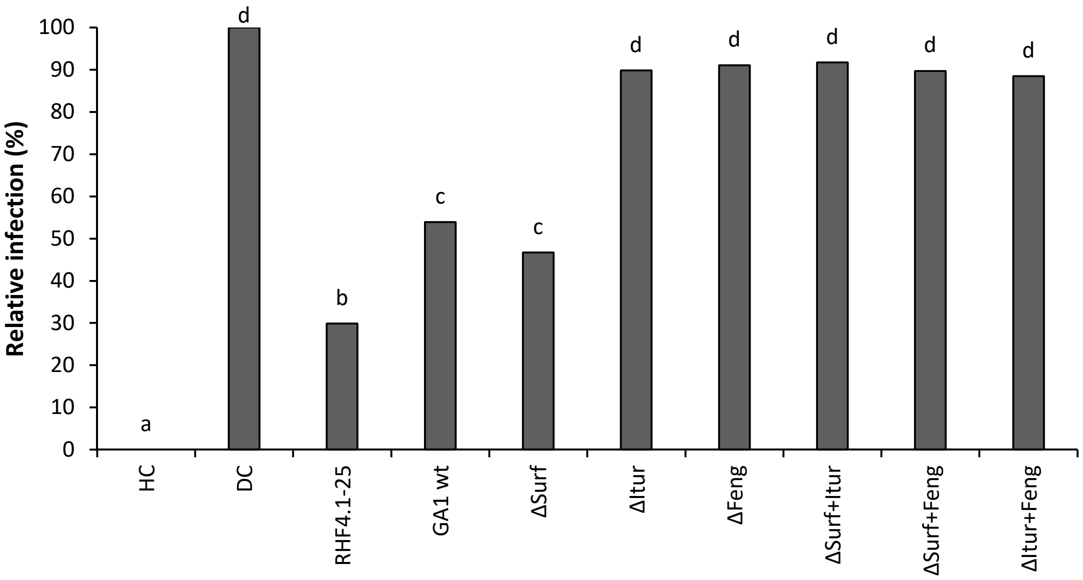


**Figure S2.** Potential of *B. velezensis* isolates RHF4.1-25 and GA1, and GA1 mutants impaired in CLiP production to induce systemic resistance against *P. oryzae* in rice (variety CO39) grown in sterile **potting soil**. Data from Figure 5 are here expressed as % relative infection in comparison with the diseased control (n=27). Univariate ANOVA followed by Duncan's post hoc tests were used and different letters among these treatments indicate statistically significant differences (P < 0.05). HC: healthy control; DC: diseased control: RHF4.1-25 and GA1: wild type *B. velezensis*;  Δ*srfaA*: surfactin mutant; Δ*ituA*: iturin mutant; Δ*fenA*: fengycin mutant. Δ*srfaA-ituA*: double mutant impaired in surfactin and iturin production;  Δ*srfaA-ituA*: double mutant impaired in surfactin and fengycin production; Δ*fenA-ituA*: double mutant impaired in iturin and fengycin production.

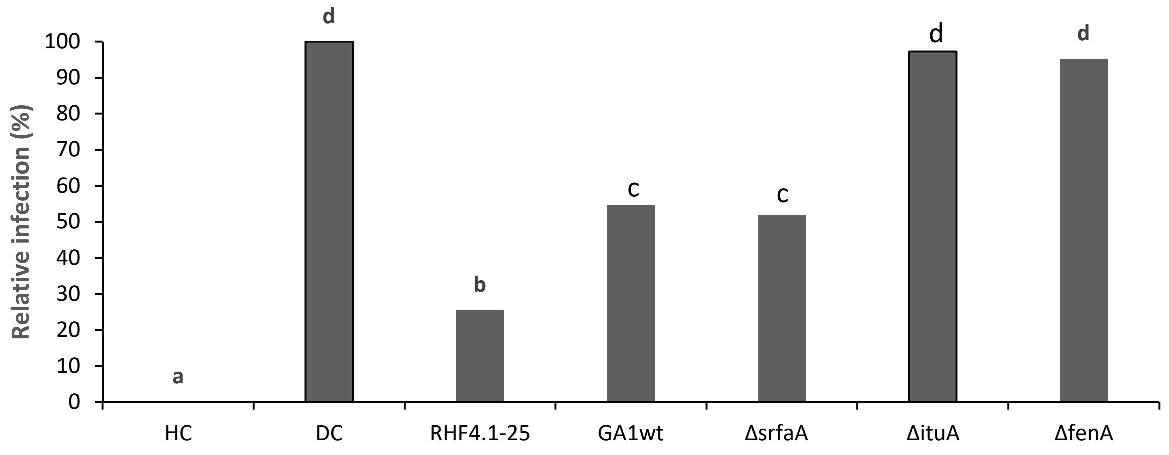


**Figure S3.** Potential of *B. velezensis* isolates RHF4.1-25 and GA1, and GA1 mutants impaired in CLiP production to induce systemic resistance against *P. oryzae* in rice (variety Jasmine 85) grown in sterile acid sulfate soil. Data from Figure 5 are here expressed as % relative infection in comparison with the diseased control (n=15). Univariate ANOVA followed by Duncan's post hoc tests were used and different letters among these treatments indicate statistically significant differences (P < 0.05). HC: healthy control; DC: diseased control; RHF4.1-25 and GA1: wild type *B. velezensis*; Δ*srfaA*: surfactin mutant of GA1; Δ*ituA*: iturin mutant of GA1; Δ*fenA*: fengycin mutant of GA1.

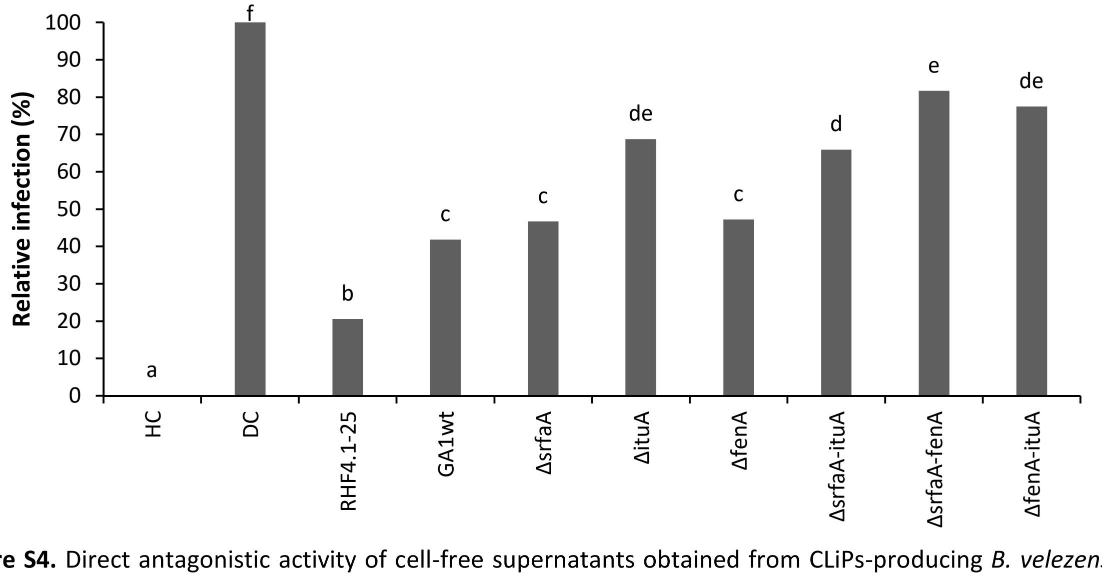


**Figure S4.** Direct antagonistic activity of cell-free supernatants obtained from CLiPs-producing *B. velezensis* RHF4.1-25, *B. velezensis* GA1 wild type and its mutants against *P. oryzae* VT5M1. Data from Figure 9 are here expressed as % relative infection in comparison with the diseased control (n=21). One-way ANOVA followed by Duncan's post hoc tests were used and different letters among these treatments indicate statistically significant differences (P < 0.05). HC: healthy control; DC: diseased control: RHF4.1-25 and GA1: wild type *B. velezensis*; Δ*srfaA*: surfactin mutant; Δ*ituA*: iturin mutant; Δ*fenA*: fengycin mutant. Δ*srfaA-ituA*: double mutant impaired in surfactin and iturin production; Δ*srfaA-ituA*: double mutant impaired in surfactin and fengycin production; Δ*fenA-ituA*: double mutant impaired in iturin and fengycin production.
